# Quinoline biodegradation by filamentous fungus *Cunninghamella elegans* and adaptive modifications of the fungal membrane composition

**DOI:** 10.1007/s11356-016-6116-4

**Published:** 2016-01-26

**Authors:** Aleksandra Felczak, Przemysław Bernat, Sylwia Różalska, Katarzyna Lisowska

**Affiliations:** Department of Industrial Microbiology and Biotechnology, Faculty of Biology and Environmental Protection, University of Lodz, 12/16 Banacha Street, 90-237 Lodz, Poland

**Keywords:** Quinoline, Degradation, Fungi, Phospholipid profile

## Abstract

Quinoline, which belongs to N-heterocyclic compounds, occurs naturally in the environment and is used in numerous industrial processes. The structures of various chemicals, such as dyes and medicines, are based on this compound. Due to that fact, quinoline and its derivatives are widely distributed in environment and can exert toxic effects on organisms from different trophic levels. The ability of the filamentous fungus *Cunninghamella elegans* IM 1785/21Gp to degrade quinoline and modulate the membrane composition in response to the pollutant was studied. *C. elegans* IM 1785/21Gp removes quinoline with high efficiency and transforms the pollutant into two novel hydroxylated derivatives, 2-hydroxyquinoline and 3-hydroxyquinoline. Moreover, due to the disruption in the membrane stability by quinoline, *C. elegans* IM 1785/21Gp modulates the fatty acid composition and phospholipid profile.

## Introduction

Quinoline, which is classified into N-heterocyclic compounds, is a pollutant widely distributed in environment. High amounts of quinoline are released during the processing of coal tar and creosote (Pereira et al. 1983; Padoley et al. 2008). Additionally, quinoline is generated during the processing of dyes, antibiotics, pesticides, or other industrial processes, and as a result, it occurs in high amounts in effluents and wastewaters (Fetzner 1998; EPA 2001; Padoley et al. 2008). Good solubility, high mobility, and persistence of quinoline cause that it is detected in both aquatic and soil ecosystems (Hartnik et al. 2007; Reineke et al. 2007; Blum et al. 2011). Moreover, quinoline and its derivatives can exert toxic effects on a variety of organisms from different trophic levels. Ecotoxicological effect of quinoline has been demonstrated toward bacteria, algae, daphnidas, and soil invertebrates (Kobetičová et al. 2011; Sochová et al. 2011). Also, genotoxic and mutagenic activities of quinoline were confirmed by Eisentraeger et al. (2008) and Neuwoehner et al. (2009). Due to that fact, increasing environmental pollution by quinoline is a worldwide concern.

Biotransformation of quinoline by bacteria is a well-known process with reference to its conditions and metabolites formed during the conversion. Various bacteria, especially those belonging to the genus *Pseudomonas*, possess the ability to tolerate and degrade quinoline (Cui et al. 2004; Zhu et al. 2008; Bai et al. 2009; Sun et al. 2009; Lin and Jianlong 2010).

On the other hand, the knowledge concerning fungal biotransformation of quinoline and the influence of the pollutant and formed derivatives on microorganisms is insignificant. Only few works describe the conversion of quinoline by fungi, which commonly occur in many environments and are extensively studied as degradation models of persistent pollutants. White rot fungi possess an ability to degrade pharmaceuticals, pesticides, and dyes (Cruz-Morató et al. 2013). Species of *Aspergillus*, *Mucor*, or *Cochliobolus* are widely used in the elimination of dangerous xenobiotics (Carvalho et al. 2011; Felczak et al. 2014; Krupiński et al. 2014). Also, fungi belonging to the genus of *Cunninghamella* are well known for their ability to metabolize various pollutants. *Cunninghamella* species transform polyaromatic hydrocarbons like phenanthrene (Pothuluri et al. 1996; Marco-Urrea et al. 2015), organotin compounds (Bernat and Długoński 2002), or drugs (Paludo et al. 2013; Ahmad et al. 2014). Additionally, as eukaryotic organisms, filamentous fungi can provide additional information about quinoline biotransformation products, their behavior, and toxicity, which are particularly important for the assessment of the ecological risk.

In this study, we assessed the abilities of *Cunninghamella elegans* IM 1785/21Gp to biotransform quinoline and specify the impact of the compound on the microorganism, taking into account especially the changes in the fatty acids and phospholipid profile and permeability of the cell membrane. To our knowledge, nothing has been yet reported on the adaptive modulation of the fungal fatty acid composition in response to quinoline.

## Materials and method

### Microorganism

The filamentous fungus, *C. elegans* IM 1785/21Gp, was obtained from the Culture Collection of the Department of Industrial Microbiology and Biotechnology, University of Lodz, Poland. The selected fungus had previously been described as a strain capable of 11β-hydroxylation of cortexolone to hydrocortisone (Długoński et al. 1997) and degradation of phenanthrene and tributyltin (TBT) (Lisowska and Długoński 1999; Bernat and Długoński 2002).

### Preparation of fungal inoculum

The spores from 10-day-old cultures on ZT slants (glucose, 4 g L^−1^; Difco yeast extract, 4 g L^−1^; agar, 25 g L^−1^; malt extract 6°Blg, up to 1 L; pH 7.0) were washed with 5-mL Sabouraud medium. The obtained spore suspension was incubated on a rotary shaker (180 rpm) at 28 °C, for 24 h. Then, the inoculum was transferred to fresh Sabouraud medium and incubated for 24 h, under the same conditions.

### Dry weight estimation

To determine the biomass production, the mycelium was separated by filtration through Whatman filter no. 1, washed twice, and dried at 105 °C to obtain constant weight.

### Elimination of quinoline

Two milliliter of the obtained preculture was transferred to 18 mL of fresh modified Czapek-Dox medium, with an appropriate amount of quinoline. Additionally, biotic controls containing fresh medium and inoculum of the examined fungus and abiotic controls consisting of fresh uninoculated media and quinoline were prepared. The initial concentrations of quinoline were in the range of 0–400 mg L^−1^. All samples were incubated on a rotary shaker (180 rpm) at 28 °C, for 48 h.

### Quinoline determination by gas chromatography–mass spectrometry and liquid chromatography–tandem mass spectrometry methods

To detect quinoline and the products of its transformation, whole samples of fungal cultures were homogenized on a FastPrep-24 Instrument and extracted twice with ethyl acetate. The collected organic phases were dried over anhydrous Na_2_SO_4_ and evaporated to dryness under reduced pressure. The samples dissolved in ethyl acetate were analyzed on a gas chromatograph Hewlett-Packard Model 6890 with a 5973 Mass Detector, on a capillary column Restek RTX-5MS (60 m × 0.25 mm × 0.23 μm). The temperature of the column for 4.5 min was 110 °C, and then, it increased to 290 °C at a rate of 20 °C min^−1^. The carrier gas was helium, at a constant flow rate of 1 mL min^−1^. The total time of analysis was 19.5 min.

For the liquid chromatography–tandem mass spectrometry (LC-MS/MS) analysis, the extracts were dissolved in methanol. A hybrid triple-quadrupole/linear ion trap mass spectrometer (3200 QTRAP LC-MS/MS, ABSciex) was used to analyze quinoline and its derivatives in fungal samples. The LC equipment included an HPLC binary solvent delivery system (Agilent Series 1200). The chromatographic separation was performed on a reversed-phase C18 column (4.6 × 50 mm, 1.8 mm Eclipse SB-C18, Agilent Technologies). The mobile phase for the LC-MS/MS analysis consisted of water and methanol; additionally, 5-mM ammonium formate was used as an additive in all solvents. The gradient profile is presented in Table 1. The volume of injection was 10 μL. Mass spectrometry of quinoline and its potential metabolites was performed using electrospray ionization (ESI), on a 3200 QTRAP system in the positive or negative ion mode. An information-dependent acquisition (IDA) method, EMS (enhanced MS)/EPI (enhanced product ion), was used to identify possible metabolites. The spectra were obtained over a range from *m*/*z* 50 to 600. Potential metabolites were also detected using the multiple-reaction monitoring (MRM) mode with a dwell time 50 ms per each ion transition. The MRM transition lists of these compounds were created based on the optimization of the obtained standards (quinoline, 2-hydroxyquinoline, 8-hydroxyquinoline, 2,6-dihydroxyquinoline, 7-hydroxycoumarin, and 3-(2,4-dihydroxyphenyl) propionic acid; Table 2). Data analysis was performed with Analyst^™^ v1.5.2 software.

**Table 1:** Linear gradient of water and methanol composition (%) for the separation of quinoline and its metabolites on a reverse-phase column (Eclipse SB-C18, 4.6 × 50 mm, 1.8 mm) coupled to an ESI/MS source

Time (min)	Water (%)	Methanol (%)	Flow rate (mL min^−1^)
0	70	30	0.6
1	70	30	0.6
5	20	80	0.6
10	20	80	0.6
10.1	70	30	0.6
12	70	30	0.6

**Table 2:** MRM transitions and MS parameters for quinoline and its potential metabolites

Analyte	MRM transition	Polarization	Retention time
Quinoline	130.1 > 103.1, 130.1 > 77.1	+	5.75
2-hydroxyquinoline	146.1 > 128.1, 146.1 > 101.1	+	4.62
8- hydroxyquinoline	146.1 > 101.1, 146.1 > 75	+	5.93
2,6-di hydroxyquinoline	162.1 > 144.1, 162.1 > 116.1	+	2.1
7-hydroxycoumarin	161 > 133, 161 > 105	–	3.98
3-(2,4-dihydroxyphenyl) propionic acid	181 > 137.1, 181 > 120.9	–	0.88

### Lipid determination

The samples were filtered on Whatman filter no. 1 and washed with distilled water twice. Then, the mycelium was homogenized with 10 mL of methanol using FastPrep-24 Instrument. After collection, the supernatant was supplemented with 2 mL of 0.9 % NaCl and extracted with chloroform. The organic phase was drained by anhydrous sodium sulfate and evaporated to dryness. The extracts were dissolved in a methanol/chloroform (4:1) mixture and analyzed. The lipid determination was performed on an Agilent 1200 HPLC system and an 3200 QTRAP mass spectrometer according to Bernat et al. (2014). All analyses were carried out on cultures containing 200 mg L^−1^ quinoline, in the stationary phase of growth.

### Permeability of fungal cell membranes

From fungal cultures incubated with or without quinoline, 1-mL samples (at least in triplicates) were withdrawn for analysis. The samples were washed twice with phosphate-buffered saline (0.1 M, pH = 7.4) and incubated with 3 mM propidium iodide in the dark, for 5 min, at room temperature. After the incubation, the samples were washed twice with PBS and 20 μL of the suspensions was mounted on a microscopic slide.

### Confocal microscopy

The images were captured using a Confocal Laser Scanning Microscope (LSM510, Zeiss) combined with an Axiovert 200M (Zeiss, Germany) inverted fluorescence microscope equipped with a Plan-Neofluar objective (40×/0.75 oil). All settings were held constant during the course of all experiments. The propidium iodide fluorescence was detected using a He–Ne laser (543 nm) and a LP filter set (560–615 nm), and the Nomarski DIC sections were also performed. The figure panels in this article represent typical results from observation.

### Statistical analysis

All experiments were carried out at least in triplicate and analyzed individually. One-way analysis of variation was used to determine the statistical significance of the results.

## Results and discussion

### Quinoline biodegradation by *C. elegans* IM 1785/21Gp

**Fig. 1:**
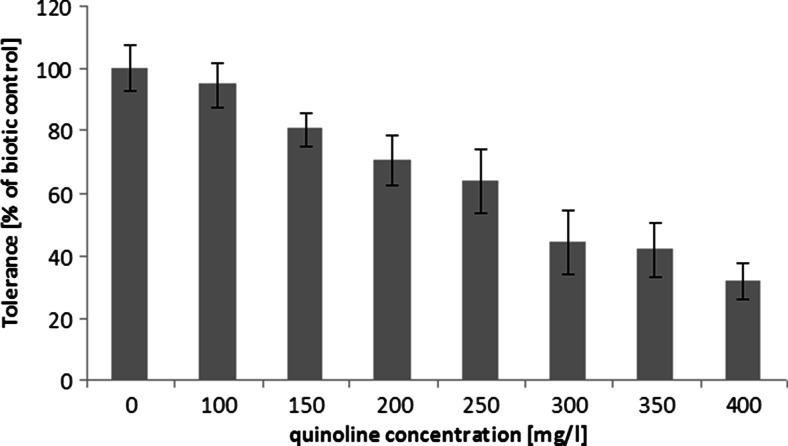
The growth of *C. elegans* IM 1785/21Gp in the presence of quinoline on mineral medium, after 48-h incubation. Data are expressed as percentage (means ± SD) of control sample without pollutant

**Fig. 2:**
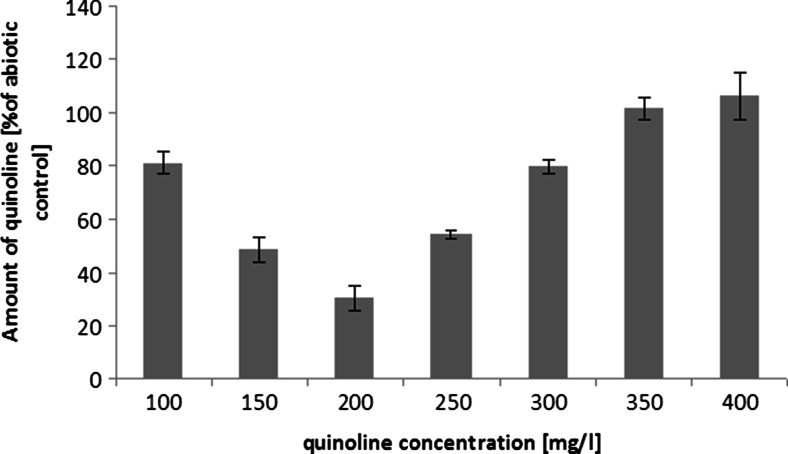
Elimination of quinoline by *C. elegans* IM 1785/21Gp after 48-h incubation on mineral medium. Data are expressed as percentage (means ± SD) of appropriate abiotic control

Preliminary stages of research included the evaluation of *C. elegans* IM 1785/21Gp growth in the presence of quinoline and the assessment of quinoline elimination. The analysis revealed that *C. elegans* IM 1785/21Gp exhibited high tolerance toward quinoline (Fig. 1). The strain was able to grow in the presence of high concentrations of the compound. The growth of *C. elegans* IM 1785/21Gp was inversely proportional to quinoline concentrations. In samples containing 200 mg L^−1^ of quinoline, the inhibition of growth reached the value of 30 % in comparison to control samples without the xenobiotic. The obtained results also indicated that the tested fungal strain possessed the ability to remove quinoline and it is connected with its high tolerance to the compound. The highest elimination of quinoline, 70 % in comparison to abiotic control, was noted in samples containing 200 mg L^−1^ quinoline (Fig. 2). Only in samples containing more than 300 mg L^−1^ of the test compound, there was no loss of the substrate, which could be associated with poor growth of *C. elegans* IM 1785/21Gp in these trials. The highest quinoline concentrations significantly affected the growth of the fungus, which in turn limited the utilization of quinoline. Therefore, the concentration of 200 mg L^−1^ was selected for the next stages of research.

**Fig. 3:**
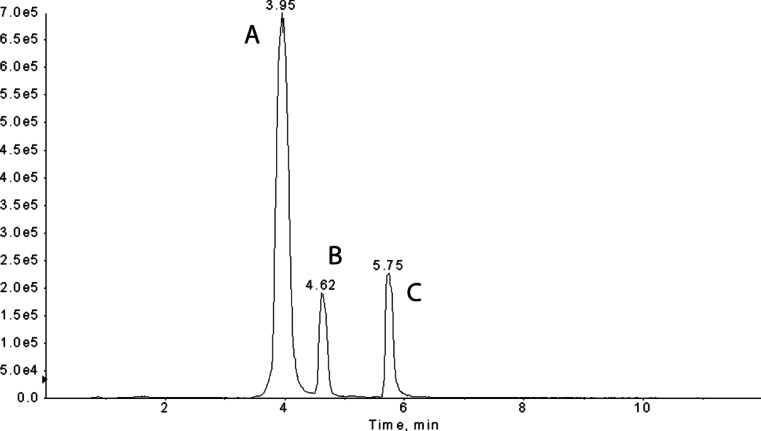
MRM scan of the extract of a 2-day-old fungal culture incubated with quinoline (200 mg/l). **a** 3-hydroxyquinoline, **b** 2-hydroxyquinoline, and **c** quinoline

**Fig. 4:**
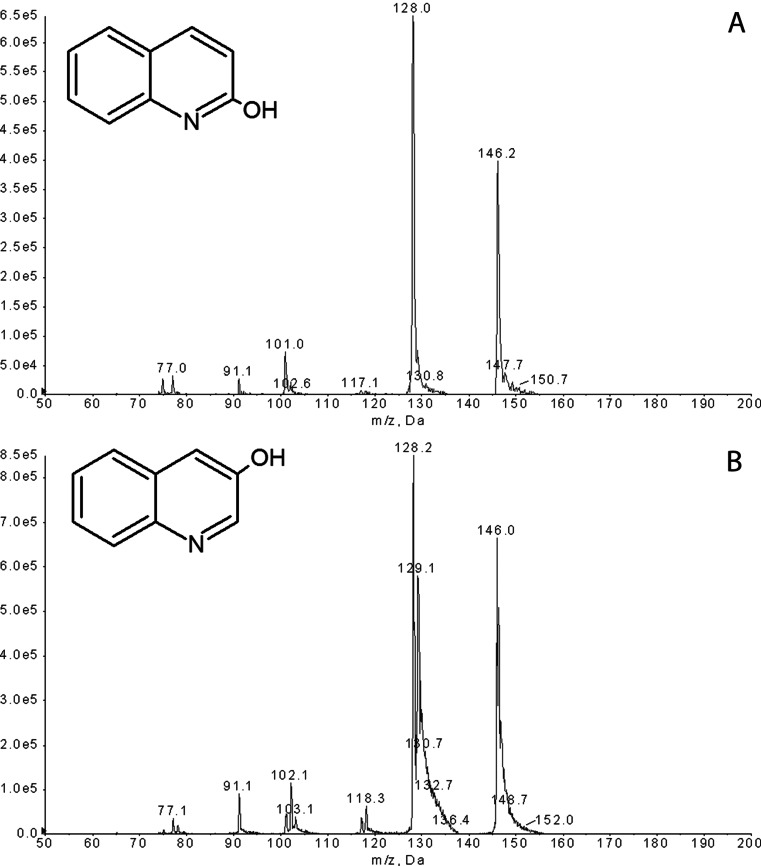
The mass chromatogram of quinoline metabolites collected at the second day of *C. elegans* IM 1785/21Gp incubation, acquired in the MRM mode **a** *m*/*z* 146.1, 2-hydroxyquinoline and **b** *m*/*z* 146.1, 3-hydroxyquinoline

Due to the fact that fungal biodegradation pathways of quinoline are poorly identified, we decided to investigate thoroughly the *C. elegans* IM 1785/21Gp ability to convert quinoline. The carried out experiments revealed that the quinoline elimination was accomplished by formation of two metabolites (Fig. 3). The analysis of these compounds was done on HPLC-MS/MS. A hybrid QTRAP mass spectrometer combines the scanning capabilities of triple quadrupole and linear ion trap instruments. In IDA methods, EMS or MRM scans trigger the acquisition of EPI spectra using EMS-IDA and MRM-IDA; quinoline (RT 5.75) and its two metabolites, 2-hydroxyquinoline (RT 4.62) and metabolite with RT 3.95, were determined in fungal samples. The increase of the 101, 117, and 128 *m*/*z* fragments by 1 in the mass spectrum of metabolite RT 3.95 in comparison to that of 2-hydroxyquinoline allowed deducing that hydroxyl group is attached to the third carbon atom (Fig. 4).

Literature data describe numerous species of bacteria with an ability to eliminate quinoline, while little is known about the possibilities of using filamentous fungi. Zhang et al. (2007) described the white rot fungus, *Pleurotus ostratus*, as a strain capable of growing in the presence of quinoline and removing the pollutant at a concentration of 250 mg L^−1^ within 15 days. The published data also indicate that *Cunninghamella* strains are able to grow and transform N-heterocyclic compounds such as carbazole (Yang and Davis 1992), quinine (Siebers-Wolff et al. 1993), 6-methylquinoline (Mountfield and Hopper 1998), benzoquinoline, and phenanthridine (Sutherland et al. 2005).

Fungal biotransformation of quinoline was presented by Sutherland et al. (1994), who showed that *C. elegans* was capable of N-oxidation of the compound. Also, *P. ostreatus* was described as a strain with an ability to convert quinoline. During the transformation process, the author detected two unidentified metabolites (Zhang et al. 2007). The production of 3-hydroxy derivatives of carbazole was indicated by Yang and Davis (1992), who revealed that *Cunninghamella echinulata* grew in the presence of N-methylcarbazole and converted it into carbazole, N-hydroxymethylcarbazole, 3-hydroxycarbazole, and 3-hydroxy-N-hydroxymethylcarbazole.

Summing up, the results presented in this work are in agreement with literature data, which indicate that strains of *Cunninghamella* are able to transform N-heterocyclic compounds.

### Quinoline impact on fatty acids and phospholipid profile of *C. elegans* IM 1785/21Gp

**Fig. 5:**
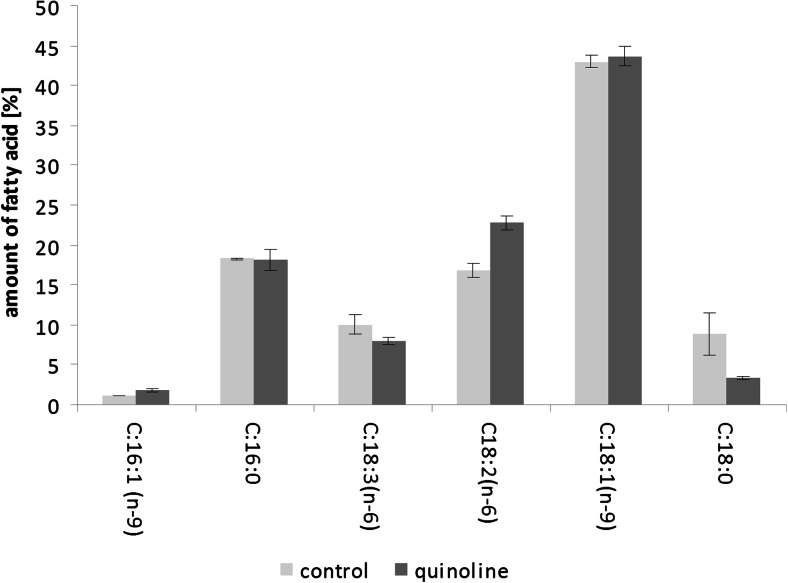
Fatty acid profile of *C. elegans* IM 1785/21Gp mycelium after incubation with or without quinoline. Data are expressed as the means ± SD

In the control samples, without the pollutant, we determined nine fatty acids in the biomass of *C. elegans* IM 1785/21Gp, but only six of them accounted for 95 % of total lipids. These were the following lipids: two saturated fatty acids, C16:0 and C18:0, and four unsaturated ones, C16:1(n-9), C18:1(n-9), C18:2(n-6), and C18:3(n-3) (Fig. 5). The detailed analysis revealed a threefold decrease in the amount of C18:0 (*P* < 0.01) in samples containing quinoline. Additionally, in the presence of the xenobiotic, an increased amount of C18:2(n-6) (*P* < 0.01) and decreased amount of C18:3(n-6) (*P* < 0.05) were observed. Corresponding results were obtained during the analysis of the phospholipid profile of *C. elegans* IM 1785/21Gp. In samples containing quinoline, decreases in the amounts of C18:1 and C18:3 were also observed. We also noted changes in the value of the unsaturated biomass index, which slightly increased from 1.07 in the control samples to 1.15 in the probes with quinoline.

Many toxic substances can interact with various components of the cell structure, so the introduction of qualitative and quantitative changes in its composition may play an important role in the adaptation mechanisms. Moreover, maintaining the adequate stability and fluidity of membrane under stress factors is essential for the proper functioning of a microorganism. This goal can be achieved by changing the saturation of membrane, composition of phospholipids, or length of fatty acids (Xia and Yuan 2009; Murínová and Dercová 2014). The decrease in the ratio of saturated fatty acids and the increase in production of unsaturated ones were shown for *Escherichia coli* incubated with ethanol (Chiou et al. 2004). On the other hand, in the presence of TBT, the amount of saturated fatty acid in *C. elegans* was found to increase (Bernat and Długoński 2007). Moreover, the mentioned mechanism is a commonly described bacterial response to the presence of toxic compounds such as chlorophenol and naphthalene (Dercová et al. 2004; Mrozik et al. 2005). In summary, the same organism may react to the presence of various xenobiotics in different ways, which can be connected with the structure of the compound, its toxicity, and mechanism of action.

**Fig. 6:**
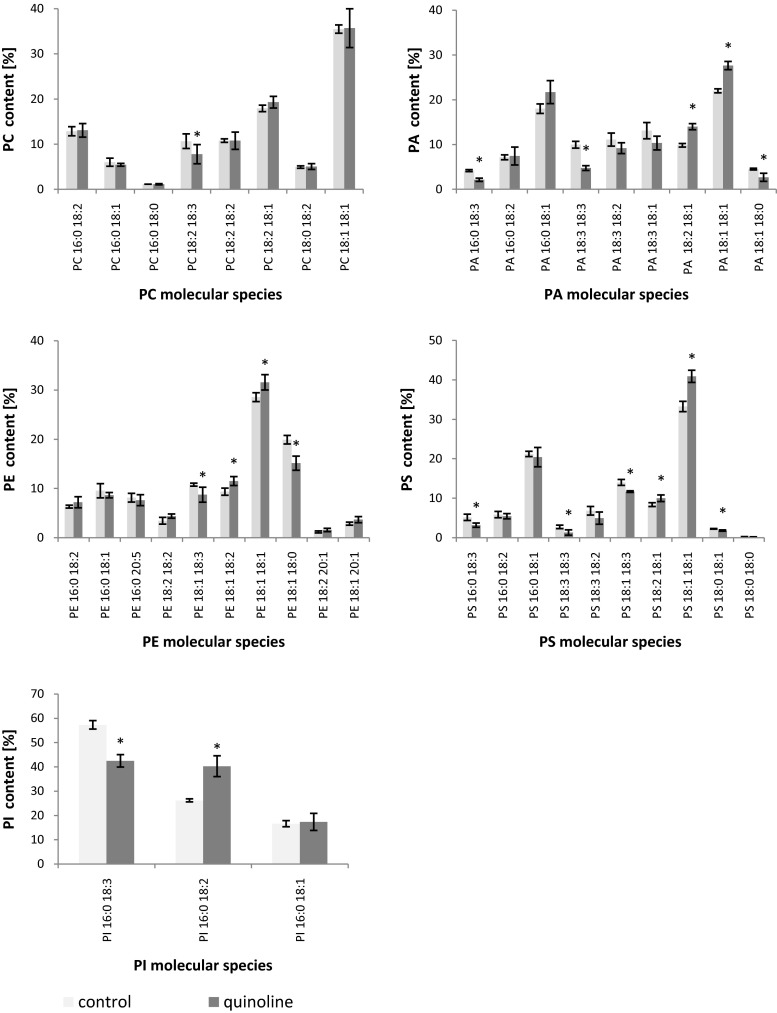
The phospholipid profile of the mycelium of *C. elegans* IM 1785/21Gp in the presence of quinoline and without addition of compound. Data are expressed as the means ± SD. *Asterisk* (*P* < 0.05) indicates values that differ significantly from the control. *PC* phosphatidylcholine, *PE* phosphatidylethanoloamine, *PS* phosphatidylserine, *PI* phosphatidylinsitol, *PA* phosphaticid acid

**Fig. 7:**
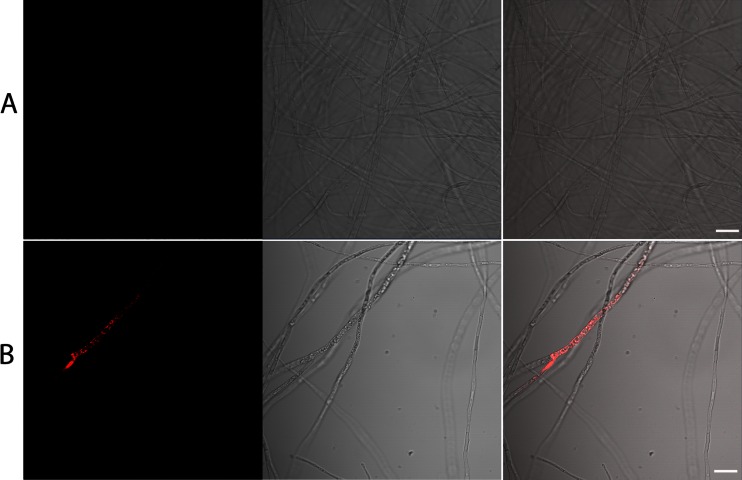
*C. elegans* IM 1785/21Gp incubated with quinoline (**b**) compared to controls without the toxic substrate (**a**). The *left panel* represents the cells stained with PI, the *middle panel* the Nomarski DIC, and the *right panel* the merged images. The *bar* represents 20 μm

In the next stage of the study, we examined the phospholipid profile of *C. elegans* IM 1785/21Gp during the incubation with quinoline. Phospholipids (PLs) can be divided to phosphatidylcholine (PC), phosphatidylethanoloamine (PE), phosphatidylserine (PS), phosphatidylinsitol (PI), phosphatidylglycerol (PG), and phosphaticid acid (PA). The analysis of the *C. elegans* IM 1785/21Gp mycelium revealed that PCs were dominant PLs and accounted for about 45–52 % of total PLs. Also, PE occurred in significant amounts reaching the value of about 37–31 %. PA, PS, and PI were presented in smaller quantities, achieving the level 3–6 % (Fig. 6 and Table 3). It is worth mentioning that the addition of quinoline caused an about 25 % increase in the PC/PE ratio in comparison to control samples. Simultaneously, with the increase in PC/PE, the unsaturated index increased from 2.3 to 2.7, for control and quinoline, respectively. Also, the analysis with propidium iodide, which stains only cells with disrupted membranes, suggested that membrane permeability of *C. elegans* IM 1785/21Gp hyphae increased in samples containing quinoline (Fig. 7). Additionally, in these samples, a 1.8-fold increase in the PA content was noted.

**Table 3:** Total amount of PLs (%) and values of characteristic index

Lipid species	Control	Quinoline
PC	45.0 ± 0.74	51.6 ± 3.28*
PA	3.1 ± 0.75	5.5 ± 0.27*
PE	37.2 ± 5.14	31.7 ± 3.21
PS	5.9 ± 0.59	4.3 ± 0.84
PI	5.4 ± 0.01	5.58 ± 0.96
PC/PE ratio	1.2 ± 0.16	1.5 ± 0.38
DBI	2.3 ± 0.21	2.7 ± 0.27

Data are expressed as the means ± SD

*PC* phosphatidylcholine, *PE* phosphatidylethanoloamine, *PS* phosphatidylserine, *PI* phosphatidylinsitol, *PA* phosphaticid acid, and *DBI* double-bond index

*P < 0.05 indicates values that differ significantly from the control

The results indicating that PC and PE are dominant PLs in the biomass of *C. elegans* IM 1785/21Gp are in accordance with the work of Bernat et al. (2014). Also, Xia and Yuan (2009) indicated that in the biomass of *Saccharomyces cerevisiae* strains, PC and PE were major lipids and accounted for 50 and 20 %, respectively.

Due to the fact, that PC is a typical bilayer lipid which stabilizes the membrane and PE promotes non-bilayer composition; their ratio could provide important information on membrane stability and integrity (de Kroon et al. 2013; Murínová and Dercová 2014). A simultaneous increase in PC/PE ratio and unsaturated index can indicate on an increase in the membrane fluidity and permeability. The disruption of membranes caused by quinoline was also confirmed by staining with propidium iodide.

PAs are used for the synthesis of other PLs, but they are also considered as signal lipids. The changes in the amount of PA may also indicate that those PLs can play a role in the tolerance to quinoline. Literature data indicated that the content of PA can increase in response to various environmental stress factors (Darwish et al. 2009; Han and Yuan 2009). Corresponding results were obtained by Bernat et al. (2014), who indicated that the addition of TBT increased the level of PA.

Toxic compounds can be collected at different membrane sites and induce changes in phospholipid profile (Murínová and Dercová 2014). Various membrane modifications enable the microorganisms to grow in the presence of pollutants. However, the data on the adaptive modulation of the filamentous fungi membrane in response to quinoline, especially on phospholipids, are limited. We suppose that the presence of quinoline disrupts the membrane stability, increasing the unsaturated lipid content, which influences the cell permeability. In order to maintain proper composition and functional behavior, the microorganism increases the production of PC and decreases the synthesis of PE.

Summing up, the presented results clearly indicate that the microscopic fungus *C. elegans* IM 1785/21Gp is able to eliminate quinoline with high efficiency. Two hydroxylated metabolites, 2-hydroxyquinoline and 3-hydroxyquinoline, are formed during the biodegradation process. Additionally, the obtained data reveal that *C. elegans* IM 1785/21Gp can modulate the fatty acids and phospholipid profile in response to quinoline. To our knowledge, this is the first report concerning the formation of novel quinoline metabolites by fungi and describing the influence of quinoline on the *C. elegans* IM 1785/21Gp membrane composition.

## Conclusions

Quinoline, which belongs to N-heterocyclic compounds, is commonly used in various industrial processes. Due to that fact, quinoline and its derivatives are widely distributed in environment and can exert toxic effects on organisms from different trophic levels. The ability of the filamentous fungus *C. elegans* IM 1785/21Gp to degrade quinoline and modulate the membrane composition in response to this pollutant was studied. *C. elegans* IM 1785/21Gp removes quinoline with high efficiency and transforms the pollutant into two novel hydroxylated derivatives, 2-hydroxyquinoline and 3-hydroxyquinoline. Moreover, due to the disruption in the membrane stability by quinoline, *C. elegans* IM 1785/21Gp modulates the fatty acid composition and phospholipid profile. To our knowledge, this is the first report on the adaptive modification of the membrane composition in *C. elegans* IM 1785/21Gp mycelium in response to quinoline.

## References

[CR1] Ahmad MS, Zafar S, Bibi M, Bano S, Atia-Tul-Wahab A-U-R, Iqbal Choudhary M (2014). Biotransformation of androgenic steroid mesterolone with *Cunninghamella blakesleeana* and *Macrophomina phaseolina*. Steroids.

[CR2] Bai Y, Sun Q, Zhao C, Wen D, Tang X (2009). Simultaneous biodegradation of pyridine and quinoline by two mixed bacterial strains. Appl Microbiol Biotechnol.

[CR3] Bernat P, Długoński J (2002). Degradation of tributyltin by the filamentous fungus *Cunninghamella elegans*, with involvement of cytochrome P-450. Biotechnol Lett.

[CR4] Bernat P, Długoński J (2007). Tributyltin chloride interactions with fatty acids composition and degradation ability of the filamentous fungus *Cunninghamella elegans*. Int Biodeterior Biodegradation.

[CR5] Bernat P, Gajewska E, Szewczyk R, Słaba M, Długoński J (2014). Tributyltin (TBT) induces oxidative stress and modifies lipid profile in the filamentous fungus *Cunninghamella elegans*. Environ Sci Pollut Res Int.

[CR6] Blum P, Sagner A, Tiehm A, Martus P, Wendel T, Grathwohl P (2011). Importance of heterocyclic aromatic compounds in monitored natural attenuation for coal tar contaminated aquifers: a review. J Contam Hydrol.

[CR7] Carvalho MB, Tavares S, Medeiros J, Núñez O, Gallart-Ayala H, Leitão MC, Galceran MT, Hursthouse A, Pereira CS (2011). Degradation pathway of pentachlorophenol by *Mucor plumbeus* involves phase II conjugation and oxidation-reduction reactions. J Hazard Mater.

[CR8] Chiou RY, Phillips RD, Zhao P, Doyle MP, Beuchat LR (2004). Ethanol-mediated variations in cellular fatty acid composition and protein profiles of two genotypically different strains of *Escherichia coli* O157:H7. Appl Environ Microbiol.

[CR9] Cruz-Morató C, Ferrando-Climent L, Rodriguez-Mozaz S, Barceló D, Marco-Urrea E, Vicent T, Sarrà M (2013). Degradation of pharmaceuticals in non-sterile urban wastewater by *Trametes versicolor* in a fluidized bed bioreactor. Water Res.

[CR10] Cui M, Chen F, Fu J, Sheng G, Sun G (2004). Microbial metabolism of quinoline by *Comamonas* sp. World J Microbiol Biotechnol.

[CR11] Darwish E, Testerink C, Khalil M, El-Shihy O, Munnik T (2009). Phospholipid signaling responses in salt-stressed rice leaves. Plant Cell Physiol.

[CR12] de Kroon AI, Rijken PJ, de Smet CH (2013). Checks and balances in membrane phospholipid class and acyl chain homeostasis, the yeast perspective. Prog Lipid Res.

[CR13] Dercová K, Čertík M, Mal’ová A, Sejáková Z (2004). Effect of chlorophenols on the membrane lipids of bacterial cells. Int Biodeterior Biodegradation.

[CR14] Długoński J, Paraszkiewicz K, Sedlaczek L (1997). Maintenance of steroid 11-hydroxylation activity in immobilized *Cunninghamella elegans* protoplasts. World J Microbiol Biotechnol.

[CR15] Eisentraeger A, Brinkmann C, Hollert H, Sagner A, Tiehm A, Neuwoehner J (2008). Heterocyclic compounds: toxic effects using algae, daphnids, and the *Salmonella*/microsome test taking methodical quantitative aspects into account. Environ Toxicol Chem.

[CR16] EPA (2001) Toxicological review of quinoline. http://www.epa.gov/iris/toxreviews/1004tr.pdf

[CR17] Felczak A, Bernat P, Długoński J (2014). Biodegradation of octyltin compounds by *Cochliobolus lunatus* and influence of xenobiotics on fungal fatty acid composition. Process Biochem.

[CR18] Fetzner S (1998). Bacterial degradation of pyridine, indole, quinoline, and their derivatives under different redox conditions. Appl Microbiol and Biotechnol.

[CR19] Han PP, Yuan YJ (2009). Lipidomic analysis reveals activation of phospholipid signaling in mechanotransduction of *Taxus cuspidate* cells in response to shear stress. FASEB J.

[CR20] Hartnik T, Norli HR, Eggen T, Breedveld GD (2007). Bioassay-directed identification of toxic organic compounds in creosote-contaminated groundwater. Chemosphere.

[CR21] Kobetičová K, Simek Z, Brezovský J, Hofman J (2011). Toxic effects of nine polycyclic aromatic compounds on *Enchytraeus crypticus* in artificial soil in relation to their properties. Ecotoxicol Environ Saf.

[CR22] Krupiński M, Janicki T, Pałecz B, Długoński J (2014). Biodegradation and utilization of 4-n-nonylphenol by *Aspergillus versicolor* as a sole carbon and energy source. J Hazard Mater.

[CR23] Lin Q, Jianlong W (2010). Biodegradation characteristics of quinoline by *Pseudomonas putida*. Bioresour Technol.

[CR24] Lisowska K, Długoński J (1999). Removal of anthracene and phenanthrene by filamentous fungi capable of cortexolone 11-hydroxylation. J Basic Microbiol.

[CR25] Marco-Urrea E, García-Romera I, Aranda E (2015) Potential of non-ligninolytic fungi in bioremediation of chlorinated and polycyclicaromatic hydrocarbons. New Biotechnol doi: 10.1016/j.nbt.2015.01.005 10.1016/j.nbt.2015.01.005 25681797

[CR26] Mountfield RJ, Hopper DJ (1998). The formation of 1-hydroxymethylnaphthalene and 6-hydroxymethylquinoline by both oxidative and reductive routes in *Cunninghamella elegans*. Appl Microbiol Biotechnol.

[CR27] Mrozik A, Łabużek S, Piotrowska-Seget Z (2005). Changes in fatty acid composition in *Pseudomonas putida* and *Pseudomonas stutzeri* during naphthalene degradation. Microbiol Res.

[CR28] Murínová S, Dercová K (2014) Response mechanisms of bacterial degraders to environmental contaminants on the level of cell walls and cytoplasmic membrane. Int J Microbiol doi:10.1155/2014/873081 10.1155/2014/873081 PMC4099092 25057269

[CR29] Neuwoehner J, Reineke AK, Hollender J, Eisentraeger A (2009). Ecotoxicity of quinoline and hydroxylated derivatives and their occurrence in groundwater of a tar-contaminated field site. Ecotoxicol Environ Saf.

[CR30] Padoley KV, Mudliar SN, Pandey RA (2008). Heterocyclic nitrogenous pollutants in the environment and their treatment options—an overview. Bioresour Technol.

[CR31] Paludo CR, da Silva EA, Santos RA, Pupo MT, Emery FS, Furtado NAJC (2013). Microbial transformation of β-lapachone to its glycosides by *Cunninghamella elegans* ATCC 10028b. Phytochem Lett.

[CR32] Pereira W, Rostad C, Garbarnio J, Hult M (1983). Groundwater contamination by organic bases derived from coal-tar wastes. Environ Toxicol Chem.

[CR33] Pothuluri JV, Evans FE, Heinze TM, Fu PP, Cerniglia CE (1996). Fungal metabolism of 2-nitrofluorene. J Toxicol Environ Health.

[CR34] Reineke AK, Goen T, Preiss A, Hollender J (2007). Quinoline and derivatives at a tar oil contaminated site: hydroxylated products as indicator for natural attenuation?. Environ Sci Technol.

[CR35] Siebers-Wolff S, Arfmann H-A, Abraham W-R, Kieslich K (1993). Microbiological hydroxylation and N-oxidation of cinchona alkaloids. Biocatal.

[CR36] Sochová I, Hofman J, Holoubek I (2011). Effects of seven organic pollutants on soil nematode *Caenorhabditis elegans*. Environ Int.

[CR37] Sun Q, Bai Y, Zhao C, Xiao Y, Wen D, Tang X (2009). Aerobic biodegradation characteristics and metabolic products of quinoline by a *Pseudomonas* strain. Bioresour Technol.

[CR38] Sutherland JB, Freeman JP, Williams AJ, Cerniglia CE (1994). N-oxidation of quinoline and isoquinoline by *Cunninghamella elegans*. Exp Mycol.

[CR39] Sutherland JB, Cross EL, Heinze TM, Freeman JP, Moody JD (2005). Fungal biotransformation of benzo[f]quinoline, benzo[h]quinoline, and phenanthridine. Appl Microbiol Biotechnol.

[CR40] Xia JM, Yuan YJ (2009). Comparative lipidomics of four strains of *Saccharomyces cerevisiae* reveals different responses to furfural, phenol, and acetic acid. J Agric Food Chem.

[CR41] Yang W, Davis PJ (1992). Microbial models of mammalian metabolism: biotransformation of N-methylcarbazole using fungus *Cunninghamella echinulata*. Drug Metab Dispos.

[CR42] Zhang X, Yan K, Ren D, Wang H (2007). Studies on quinoline biodegradation by a white rot fungus (*Pleurotus ostreatus* BP) in liquid and solid state substrates. Fresenius Environ Bull.

[CR43] Zhu S, Liua D, Fana L, Nia J (2008). Degradation of quinoline by *Rhodococcus* sp. QL2 isolated from activated sludge. J Hazard Mater.

